# Extensive usage of insecticide and changing crop rotation patterns: A South Dakota case study

**DOI:** 10.1371/journal.pone.0208222

**Published:** 2018-11-29

**Authors:** Scott Fausti, Deepthi E. Kolady, Evert Van der Sluis, Jonathan Lundgren, Bashir A. Qasmi

**Affiliations:** 1 College of Business, California State University, Monterey Bay, California, United States of America; 2 Department of Economics, South Dakota State University, Brookings, South Dakota, United States of America; 3 Ecdysis Foundation, Blue Dasher Farm, Estelline, South Dakota, United States of America; College of Agricultural Sciences, UNITED STATES

## Abstract

Driven by factors such as an increased reliance on genetically modified crops, government policies, and market forces, the crop mix in South Dakota and elsewhere in the United States has become less diverse and moved toward the production of corn and soybeans as the most predominant cash crops over the past two decades. Coinciding with a reduced complexity of crop rotation practices, the prevalence of mono-cropping has increased and crop chemical usage has changed as well. Overall, the reduced reliance on traditional crop rotation practices for mitigating pests corresponds with an increase in crop acres treated with insecticides, expressed as a proportion of total cropland acres, and referred to in the literature as the extensive usage of insecticides. In this paper, we identify how changing cropping patterns in South Dakota have affected the extensive usage of insecticides, an aspect often overlooked by producers and policy makers. Results indicate that increased corn production has contributed to an increase in the share of cropland acres treated with insecticides at the county level in eastern South Dakota.

## Introduction

The agricultural sector in South Dakota, consistent with national trends, has undergone considerable change over the past several decades [1]. Structural changes are in part reflected in environmental changes, including landscape simplification in the form of a move toward production system homogeneity. In crop farming, this is reflected in increasingly simple crop rotations. While it is difficult to identify the importance of individual drivers of crop rotation pattern changes, possible factors include the increased reliance on technology (adoption of genetically modified [GM] seed, drainage tile modification to cropland, and computer aided field crop production), federal and state policies (in the form of biofuel mandates and various farm bill programs), and market forces (such as high corn and soybean prices relative to those of other crops). The increased share of acres planted to GM crop varieties and the rapid expansion of corn-based ethanol production contributed to a movement away from traditional crop rotation practices toward a system with reduced crop diversity dominated by corn and soybeans [2–5].

In this study, we adopt a mixed modelling approach to analyze Agricultural Census data to examine whether the increase in corn and soybean acres at the expense of wheat and hay acres have contributed to the increase in extensification of insecticide use that is defined as an increase in the acres of land treated with insecticides, in South Dakota. An empirical linkage is established between the increase in corn and soybean acres and the increase in cropland acres treated with insecticides in South Dakota. This linkage raises questions about the long-term relationship between an agricultural production system dominated by a corn/soybean rotation pattern and the extensive use of insecticide.

### Cropping patterns in Eastern South Dakota

In relative terms, both corn and soybean acres have increased in recent Census years. In particular, the share of corn acres in South Dakota increased from 29.3% to 36.9% of total cropland acres between in 2002 and 2012. The share of soybean acres planted increased even more dramatically and consistently over the past four decades, from a mere 3.3% in 1978 to 30.1% of total cropland acres in 2012. Only in 2007, did the upward trend in the proportion of soybean acres between Census years, experience a temporary decline to 23.4% of total cropland acres in 2007.

**Fig 1:**
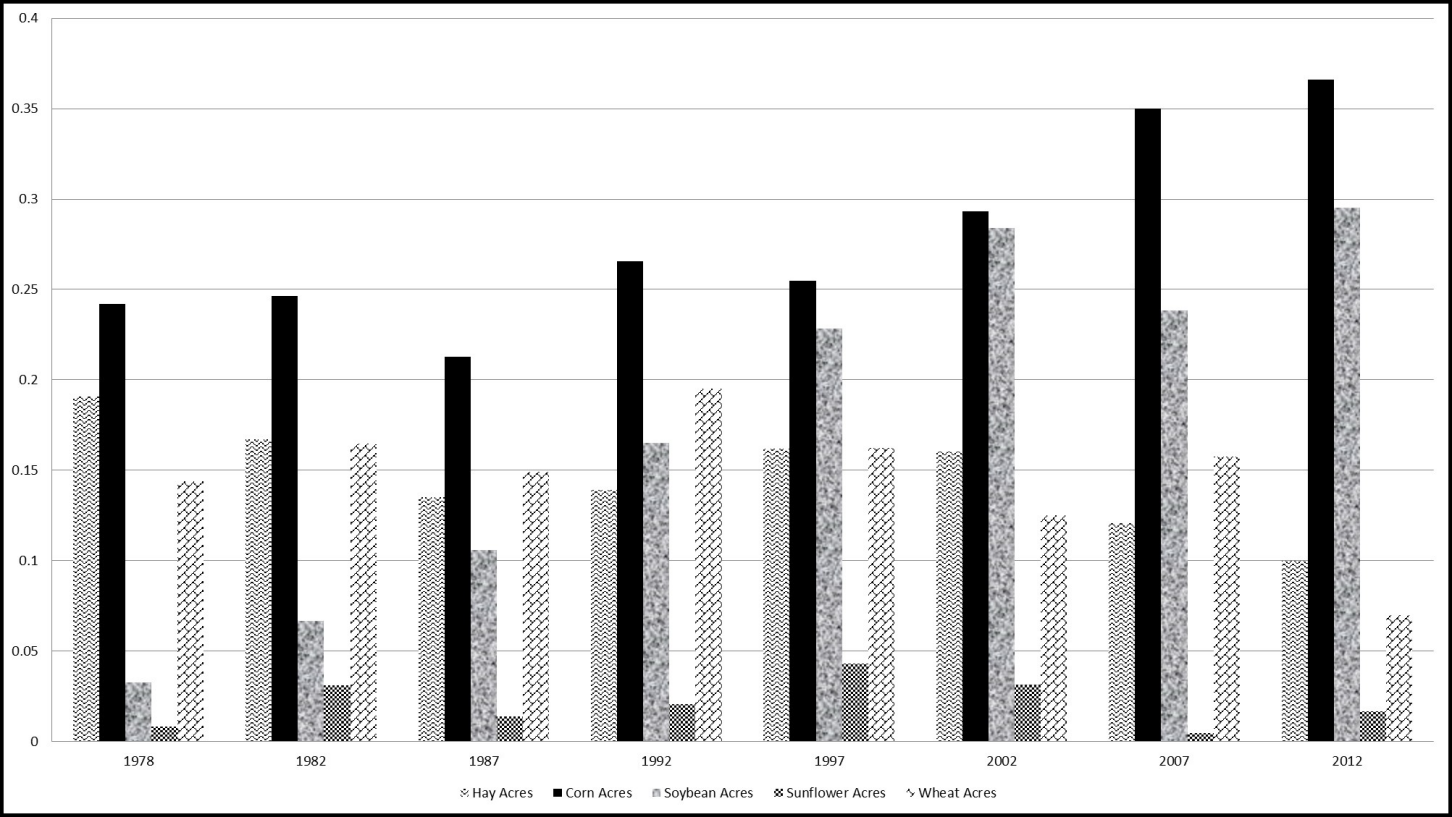
Proportion of total crop acres—East River South Dakota.

Fig 1 shows that while the share of wheat acres planted remained stable at an average of 15.6% of total cropland acres between Census years 1978 and 2007, it declined to 6.6% of total cropland acres in 2012. Hay acres harvested averaged 19.9% of total cropland acres from 1978 to 2002 but dropped to 12.2% and 9.8% of total cropland acres, respectively, in the two most recent Census reporting years. The large decline in wheat and hay acres in 2012 (as a proportion of total cropland acres) coincides with the 2012 expansion in acres planted to corn.

### Chemical usage in row crop production

**Fig 2:**
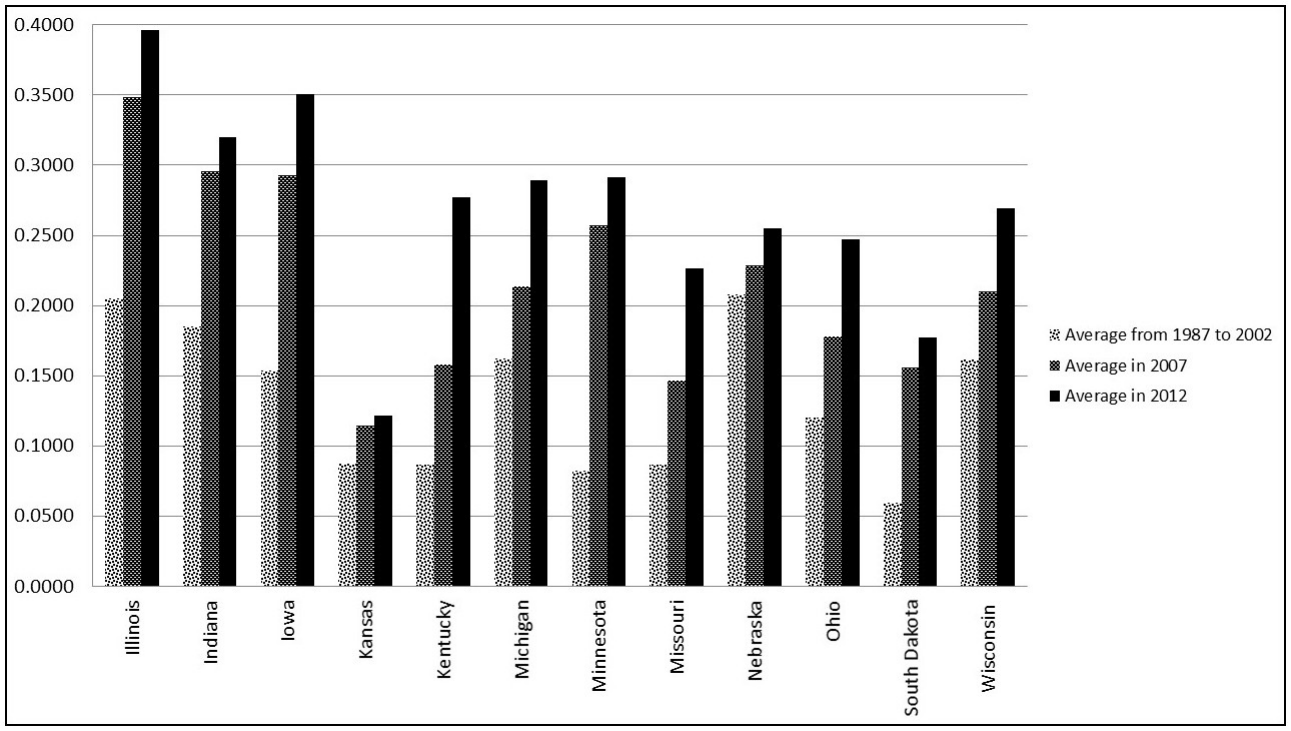
Proportion of acres treated with insecticide for Midwestern states.

**Fig 3:**
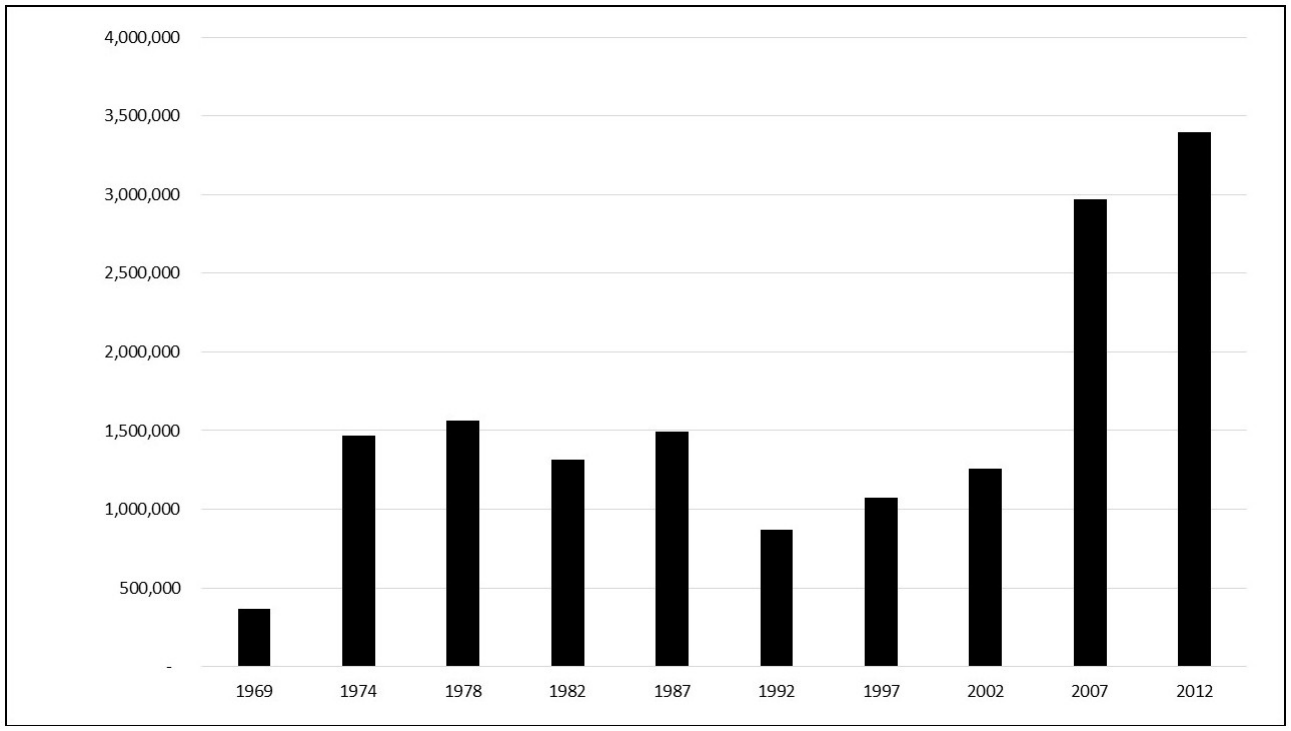
South Dakota cropland acres treated with insecticides.

While the intensive use of insecticides (measured in terms of average pounds per acre applied) has declined [6–8], the extensive use of insecticides (measured by the proportion of crop acres treated) has increased [3, 9] since the introduction of the GM technology in the 1990s, particularly over the past two Agricultural Census periods (Fig 2). For example, the number of acres treated with insecticides remained stable at an average of 1.2 million acres (5.9% of total cropland acres) over the six Agricultural Census years between 1974 and 2002, but they nearly tripled to 2.9 and 3.2 million acres (15.6% and 17.8% of total cropland acres), respectively, in Census years 2007 and 2012 in South Dakota (Fig 3). Although these trends do not establish a causal relationship, the positive correlation between the growing number of acres treated with insecticides and the increased reliance on GM crops in South Dakota suggests that the extensive use of insecticides rose (additional acres treated) and the ecosystem’s exposure to insecticides broadened over the past two Agricultural Census periods. Over the past two decades U.S. crop production has experienced simultaneous trends of: a) a widespread adoption of GM technology, b) a positive trend in weed resistance to glyphosate, c) a rising corn rootworm resistance to Bt-derived toxins, d) an increase in the emergence of non-target pests, e) an increase in the use of seed treatments, and f) an expansion in row crop acres together with a reduction in traditional crop rotations practices. Perhaps even more important than establishing a causal relationship between these overall trends is the question of whether the overall ecosystem health will be able to support the long-term sustainability of agriculture production in its current form. If an increase in extensive use of insecticides increases the ecosystem exposure to insecticides, it potentially will have negative implications for the long-term sustainability of agriculture production system and overall ecosystem health.

The increased demand for ethanol and biodiesel, coinciding with an increase in crop prices during the first decade since 2000, contributed to the conversion of marginal grassland areas into corn and soybean production [10]. Expanding corn and soybean plantings to areas with relatively unfavorable production conditions may lead to periods of plant stress, subject the crops to unduly non-target pest exposure, and cause agricultural producers to take countermeasures in the form of insecticide treatments.

Unlike cotton, which traditionally has been subject to intensive insecticide usage, insecticide application to corn has generally been relatively modest, mainly because insecticide treatments were considered ineffective for controlling pests such as corn stalk borers [7]. However, the introduction of GM technology led to increases in seed stock costs and insecticide treatment of seed. The latter is a relatively inexpensive approach to protecting seed from non-target pests and thus ensuring the efficient use of the seed [9]. In particular, pre-emptive use of neonicotinoids as seed treatments has resulted in an increase in the number of cropland acres treated with insecticides, a factor not captured in national pesticide surveys [9]. While per-acre insecticide use is on the decline even after accounting for the increased use of neonicotinoids, this does not take into account chemical toxicity [8].

Over time, the heavy reliance on GM crops also led to weed resistance to glyphosate, and to the emergence of corn rootworm populations resistant to the insecticidal toxins derived from the *Bacillus thuringiensis* (Bt) bacterium expressed in several corn hybrids [11, 12]. In turn, corn rootworm resistance development contributed to increased use of soil-applied (at the time of planting) insecticides [6].

### Previous empirical work on insecticide use

Chemical usage application decisions in crop production are complex, not only because detailed data regarding specific chemicals are lacking, but also because chemical treatment application decisions depend on a variety of factors. Many of these complicating factors are outside of the purview of agricultural producers, such as the pesticide price and effectiveness, the degree and spread of pest infestation levels, the crop maturation stage, and weather-related factors. Other factors may to some extent be within the control of the manager, including alternative technical and managerial approaches to mitigating pest damage, pest mitigation timing, the quality of seed varieties planted, and the effectiveness of the chemical compounds used to treat pest infestations.

A key determinant of insecticide use is the (perception of the) presence of harmful insect pests. While the amount of insecticides applied is generally a function of the harmful insect pest infestation level, the latter may be difficult to predict, vary from year to year, and spread rapidly beyond critical thresholds. Hence, uncertainty surrounding the potential for future pest infestation problems can lead producers to apply insecticides preventively, as a form of insurance against pest infestations that may develop [13, 14].

A variety of modeling techniques have been used to capture GM crop adoption and insecticide usage decisions. Early studies on GM crop adoption decisions employed two-stage least squares regression modeling techniques to capture the simultaneity of the GM crop adoption decision and the use of insecticides. The underlying modeling assumptions were that perceived pest pressures influence the decision of whether to plant GM varieties and how much to spray [15, 16]. However, the near complete adoption levels of GM crops in South Dakota render such models ineffective for assessing the ex-post effect of widespread GM crop adoption on the number of acres treated with insecticide as a proportion of total cropland acres.

A common approach to empirically assessing insecticide use in crop production is to analyze the volume or weight of insecticide applied [6, 16–18]. However, because this approach does not account for critical factors such as chemical toxicity, concentration, and application practices, results of such studies are not comparable for different crop chemicals. To address this concern, [8] weigh pesticides by their environmental impact quotient and show that U.S. per acre insecticide use has declined for both corn and soybeans, based on their analysis of a unique and proprietary farm-level data set.

An alternative method for assessing a change in insecticide usage is to evaluate the change in the trend of acres treated to acres planted. Available evidence (3) suggests that changing cropping patterns in South Dakota affected insecticide usage during the period from 1978 to 2007. This alternative approach of examining extensive usage of insecticides (as measured by the proportion of cropland acres treated) rather than the intensive insecticide usage (as quantified by the amount of insecticide applied per acre) [6, 8, 18] provides an alternative view of how changing crop rotation patterns have effected insecticide usage rather than relying solely on empirical studies focused on intensive insecticide usage.

## Materials and methods

### Data collection

The empirical approach employed in this study involves estimating changes in the use of crop chemicals as a percentage of total cropland acres treated with insecticides in eastern South Dakota. Secondary county-level data were collected to empirically measure the change in the extensive use of insecticide in eastern South Dakota. Crop-specific acres planted at the county level were taken from the National Agriculture Statistics Service [19], and county-level data on total cropland acres and acres treated with crop chemicals were collected from the U.S. Census of Agriculture from 1978 to 2012. The latter source reports acres treated at the county level, but not by crop. To overcome the lack of crop-specific chemical usage at the county level, the USDA definition of the “proportion of acres treated out of total cropland acres at the county level”, is used as a generic empirical proxy for extensive insecticide usage.

Discussions with corn and soybean producer groups in the state suggest that the emergence of soybean aphids in recent years was a key contributing factor of the increase in the acres treated with insecticides. This suspicion, combined with the fact that comprehensive data on pest infestations by county do not exist, led us to focus on soybean aphid infestations as the key pest infestation indicator to capture the spread of insect pest infestations across South Dakota. Aphid data on South Dakota county-level infestations are collected by way of field surveys conducted by entomologists. Although it has been reported [2] that 46 out of 66 South Dakota counties had soybean aphid infestations in 2007, only six out of 44 counties in eastern South Dakota did not have a soybean aphid infestation in 2009 [20]. However, these six counties east of the Missouri River (Faulk, Hand, Hughes, Hyde, Porter, and Walworth) did not plant soybeans in 2009 [19], suggesting all South Dakota counties east of the Missouri River with soybean plantings also had aphid infestations in 2009. Hence, in our modeling approach described below, we assume that for counties with soybean plantings, aphid = 1, and otherwise aphid = 0 for Census year 2012.

The dataset includes county-level observations for all 44 eastern South Dakota counties over the past eight Census years (1978–2012), resulting in 352 observations. The data include acres planted with corn, soybeans, wheat, and sunflower, as well as hay acres harvested. Jointly, these crops accounted for 62% and 86% of total cropland in 1978 and 2012, respectively, in eastern South Dakota. Harvested acres were used rather than acres planted to represent the production of hay, because each hay acre planted may be harvested multiple years. The constructed panel dataset contains no missing observations for any counties over the observed years, making it a balanced data set [21].

### Econometric models and analyses

All of South Dakota’s main agricultural crops (corn, soybeans, wheat, sunflowers, hay, and other small grains) have significant pest problems and are thus prone to suffer from yield reduction. Because data on the magnitude of pest infestations are not available at the South Dakota county level, the application of ordinary least squares (OLS) for analyzing these county-level data would suffer from omitted variable bias. Instead, a mixed-model approach that allows for incorporating both fixed and random effects into the model for analyzing insecticide usage at the South Dakota county level was adopted.

The adoption of a mixed modeling approach overcomes a shortcoming by [2], who ignored variation by county in the extensive usage of insecticides due to differences in, for instance, growing degree days, soil types, GM crop adoption levels, and insect populations. Thus, the use of a random effects component in our modeling techniques enables us to analyze differences in insecticide treatment by county. Additionally, the data series has been extended by adding a year of Census data. The current research also utilizes acres planted by crop as a share of total cropland acres by county as the measure of the proportion of each specific crop planted. This is in contrast to the previous study [2], which used planted acres by crop as a share of total acres planted at the county level. This revised approach is consistent with the United States Department of Agriculture (USDA)’s method for reporting acres treated with insecticides, expressed as a proportion of total cropland acres, and provides a conservative estimate of the independent variables.

#### Empirical model

A panel data regression model was constructed to investigate the influence of crop rotation patterns on the proportion of acres treated with insecticides at the county level in eastern South Dakota (44 counties). The dependent variable (PCTI) is defined as the proportion of acres treated with insecticides in a specific county. The modeling approach entailed regressing PCTI on the proportion of acres planted in a county for the following crops: corn, soybeans, sunflowers, wheat, and hay. Dummy variables (CensDum) were constructed for Census years 2007 and 2012 to capture the effects of U.S. biofuel policy initiatives and historically high corn and soybean prices in South Dakota, respectively, which may have provided an additional incentive for producers to increase insecticide usage as an insurance instrument during periods of high crop prices. South Dakota crop prices reached historical highs in 2012 (the corn price was $6.72 per bushel, and the soybean price was $14.20 per bushel), which provided a significant incentive for farmers to protect crops from insect damage. Also included is a dummy variable representing soybean aphid infestations (Aphid) by county, to capture the spread of soybean aphid infestations across South Dakota counties.

The standard assumptions associated with the linear mixed-model (LMM) are listed in Eqs 1–4. The standard vector notation is provided in the SAS/Stat 9.3 User Guide [22]. The general structure of the model is defined as:

PCTI=Χβ+Zγ+ε, (1)

γ∼N(O,G), (2)

ε∼N(O,R), (3)

COV(γ,ϵ)=0, (4)

where *PCTI* denotes the vector of dependent variable observations; matrix *X* is the design matrix associated with *β*, which represents the vector of unknown fixed effects parameters; and matrix *Z* is the design matrix representing the vector of unknown random effects parameters and associated with *γ*. The error term, *ε*, reflects an unknown random error vector. Eq 4 states that *γ* and *ε* are independent, implying that the variance of *PCTI* [23] can be defined as:

$$VAR\left[VBP\right]=ZGZ^{T}+\;R$$ (5)

The superscript notation “*T*” denotes the transpose matrix operation. *G* and *R* are the covariance matrices associated with *γ* and *ε*, respectively. The mixed procedure in SAS requires the covariance matrices *G* and *R* to be specified. Based on regression diagnostics, we assume a variance components specification for *G*, and a blocked (subject-dependent) first-order autoregressive specification for *R*.

#### Base model design

A mixed model with both fixed and random effects was used to analyze the data. The fixed-effect component of the model is appropriate for analyzing non-experimental data characterized by their lack of a scientific control group, because it allows for treating each observation as its own control [24, 25]. In the case of crops, these factors may include geographical location and technology adoption. Factors, such as, latitude, longitude, soil type, and cultural norms. The latter captures county within-effects that may include local size of farming operations, local weather conditions, pest infestation levels, producer perceptions of market conditions, and local zoning and land use restrictions. Eq 1 provides the general functional form for a Mixed Effects model. Eq 6 reflect the base model.

$${\text{PCTI}}_{\text{it}}{=\text{a}}_{\text{i}}+\text{a}+\sum_{\text{j}=1}^{\text{5}}{\text{b}}_{\text{j}}{\text{C}}_{\text{ijt}}{+\text{b}}_{\text{6}}{\text{CensDum07}}_{\text{it}}{+\text{b}}_{7}{\text{CensDum12}}_{\text{it}}\text{+}\sum_{\text{k}=1}^{\text{3}}{\text{d}}_{\text{ik}}{\text{Z}}_{\text{ikt}}{\text{+f}}_{\text{1}}{\text{Aphid}}_{\text{it}}{\text{Pctsb}}_{\text{it}}{+\text{e}}_{\text{it}}\text{,}$$ (6)

where i = 1 to 44 denotes counties, j = 1 to 5 denotes wheat, hay, sunflowers, corn, and soybeans, k = 1 to 3 denotes random effect variables for Censdum07, CensDum12, and Aphid, and t = 1 to 8 denotes the eight Census periods.

*C*_*ij*_ denotes the proportion of individual crop acres planted (*j*) to “total cropland” in each county *i*. Z_ikt_ denotes the random effect variables (*k*) in each county (*i*) at time (*t*). CensDum07 and CensDum12 are the Census-year dummies and the dummy variable *Aphid*_*it*_ has a value of one when a county was determined to be infested with soybean aphids, and zero otherwise.

The parameters *a* and *b*_*ij*_ denote the fixed effect intercept and fixed effects parameters, respectively. Parameters *b*_*6*_, and *b*_*7*_ are the effect of Census year dummy variables, and parameter *f*_*1*_ represents the interaction fixed effects parameter estimate for the interaction between the proportion of soybeans planted (*pctsb*) and aphid infestation at the county level. Parameters *a*_*i*_ and *d*_*ik*_ denote the estimated random effects. The error term *e*_*it*_ is specified in Eq 3.

The regression base model includes four random effect variables (intercept, CensDum07, CensDum12, and Aphid). Fixed effects include all five crop variables, an interaction term between the share of total cropland acres planted to soybeans and soybean aphid infestation levels, and two Census dummy variables (CensDum07 and CensDum12) to capture possible structural changes in pesticide usage that occurred during the two most recent Census years relative to previous Census years analyzed. Specifically, dummy variables for 2007 and 2012 were included to capture the near three-fold increase in insecticide usage at the State level for the most recent two Census years relative to levels observed during the previous three decades.

We assume pest populations are not correlated with crop choice, because agricultural producers can implement protective measures in case of pest infestations, and because crop choice is largely determined by market conditions. If crop choice were determined by the likelihood of pest infestation, then this would violate the assumptions needed to ensure unbiased estimation.

#### Expanded interaction term model design

To further explore the issue of the increase in the extensive usage of insecticide in eastern South Dakota during the two latest Census of Agriculture years, additional interaction terms were constructed using the Census dummy and the crop variables. The objective of including interaction terms is to capture the change in the slope relationships between the share of acres treated and the share of acres planted by crop for these two Census years relative to previous years.

Eq 6 is modified by including interaction terms for crop (*j*) and Census year dummy variables "CensDum07” and “CensDum12" to generate the expanded interaction term model in Eq 7.

$$\;\begin{array}{l} PCTI_{it}=\alpha_{i}+\;\alpha +\Sigma_{j=1}^{5}\beta_{j}\;C_{ijt}+\beta_{\text{6}}PCTC_{it}\;CensDum07_{it}\text{+}\beta_{\text{7}}PCTC_{it}CensDum12_{it}\text{+}\beta_{8}PCTW_{it}CensDum\;{\text{07}}_{it}\\ +\beta_{9}PCTW_{it}CensDum{\text{12}}_{it}\text{+}\beta_{10}PCTSF_{it}CensDum{\text{07}}_{it}\;+\beta_{11}PCTSF_{it}CensDum{\text{12}}_{it}\;+\;\beta_{12}PCTHay_{it}CensDum{\text{07}}_{it}\\ +\beta_{13}PCTHay_{it}CensDum{\text{12}}_{it}\;+\Sigma_{\text{k}=1}^{3}\gamma_{\text{ik}}{\text{Z}}_{\text{ikt}}+\theta_{\text{1}}Aphid_{it}\;Pctsb_{it}+\varepsilon_{it}\text{,} \end{array}.$$ (7)

where i = 1 to 44 denotes counties, j = 1 to 5 denotes crops wheat, hay, sunflowers, corn, and soybeans, k = 1 to 3 and t = 1 to 8.

*C*_*ij*_, Z_ikt_, Census-year dummies, and the dummy variable *Aphid*_*it*_ are all defined above. Parameters *α* and *β*_*ij*_ denote the fixed intercept and fixed-effects parameters, respectively. Parameters *β*_*6*_ to *β*_*13*_ represent interaction fixed-effect estimates for crop/Census dummy interactions. The interaction model excludes the soybean/Census dummy interaction term to address the issue of multicollinearity. Parameter *Θ*_*1*_ denotes the interaction fixed effects parameter estimates for the interaction between the proportion of soybeans planted (*pctsb*) and aphid infestation at the county level. The aphid dummy variable was dropped in both models due to multicollinearity between the aphid dummy variable and its interaction term with *pctsb*. Parameters *α*_*i*_ and *γ*_*ik*_ denote the estimated random effects.

The interaction model includes the five crop variable fixed effect covariates; four interaction term covariates for the interaction between corn, hay, sunflowers, and wheat with the Census year dummy variables; and the soybean-aphid interaction term. The random effect part of the model includes a random intercept, and three random dummy variables; CensDum07, CensDum12, and Aphid. The associated parameter estimates are: *a*_*i*_, *α*_*i*_, *d*_*ik*_, and *γ*_*ik*_, respectively. These random-effect covariates capture the unique random attributes (disturbances) occurring within counties. When a covariate is used as both a fixed effects and random effects, then the estimated fixed effect parameter is interpreted as the average effect of the covariate across subjects and the random effect parameter is marginal with-in subject effect of the covariate.

#### Model diagnostics

The empirical analyses employed SAS version 9.2. The SAS code is provided in S1 File. The mixed-effects model procedure used SAS’s Restricted Maximum Likelihood method. The LMM procedure in SAS provides great flexibility when dealing with regression diagnostic issues [22].

**Fig 4:**
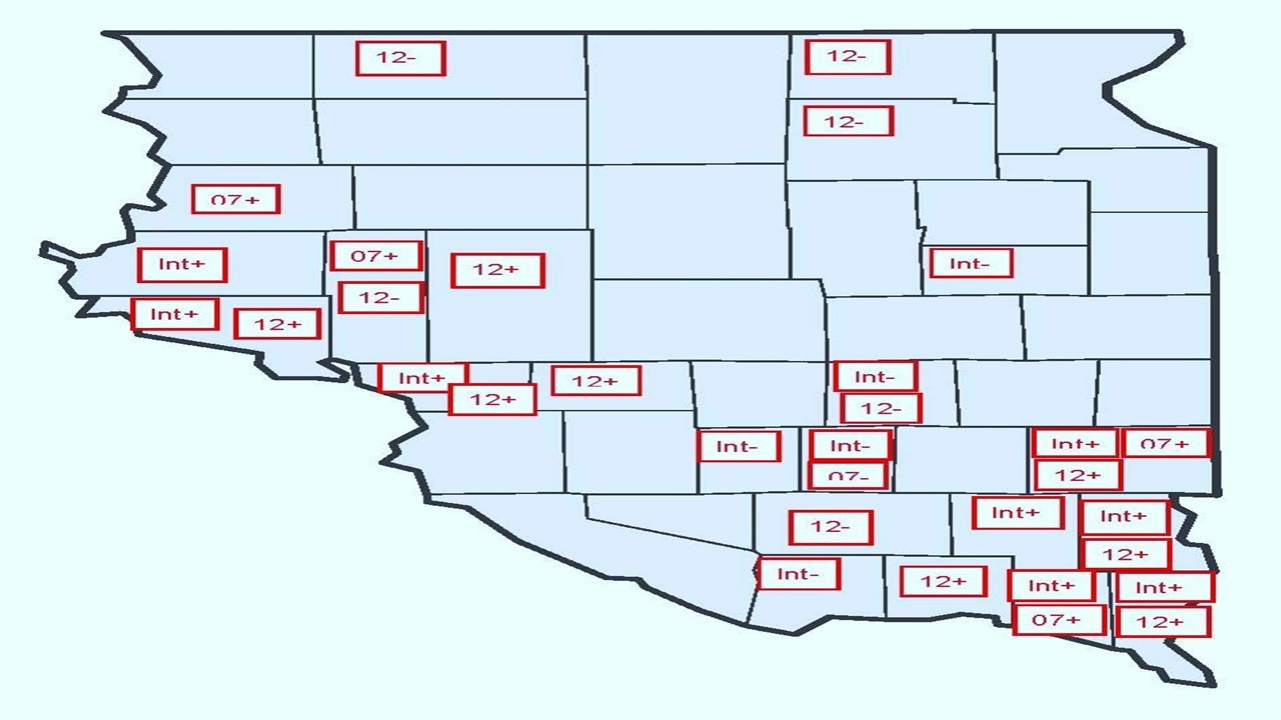
Forty-four eastern South Dakota counties with statistically significant county random coefficients displayed. Note: Symbols (+) and (–) indicated parameter sign.

The first diagnostic issue is the use of a “sandwich estimator” approach. This approach estimates robust standard errors associated with parameter estimates [22]. One of the advantages of the mixed model approach in SAS is the option of generating the estimated random effect coefficients (*d*_*ik*_ and γ_ik_). Fig 4 plots the statistically significant random effect coefficients by county that are estimated using the base model. Statistically significant coefficients (p-value < 0.10) reflect counties where the random effects coefficients measure the difference between the average PCTI of county *i* and the average PCTI value for all 44 counties with respect to the four random effects variables. For example, the fixed effect estimate for CensDum07 in the base model represents the average effect of Census year 2007, and the statistically significant random effects coefficients *d*_*ik*_ reflect the marginal adjustment to the fixed effect estimate for county *i*. Clustering of statistically significant coefficients shown in Fig 4 suggests that the use of the sandwich estimator approach is justified.

The default covariance structure for the mixed procedure is variance components. The variance components structure was selected for matrix G, and the autoregressive of order one structure was selected for matrix R. Mixed versus fixed effect assumptions along with covariance structure assumptions were based on the Null Model Likelihood Ratio Test. The SAS mixed procedure does not have a Hausman option for testing fixed vs. random effects. To confirm that the empirical results are not affected by endogeneity bias, correlation analyses were conducted between the residuals and independent variables. The null hypothesis of *corr(x*,*ε) = 0* was confirmed for all covariates (P-value > 0.10) and all correlation coefficients were smaller than 0.03.

Table 1 provides information on the key variables used in the empirical model, and Table 2 lists their summary statistics. The mixed model procedure in SAS allows for conducting variance components analysis and confirms the validity of selecting a mixed modeling approach and the use of a serial correlation correction procedure. Empirical verification of the decision to use variance components analysis for the two estimated regression models discussed above is provided as supporting information in S1 and S2 Tables.

**Table 1:** Key variables used in the empirical analysis.

Variable Name	Definition	Source/Description
**pcti**	Dependent variable: % of total planted acres treated w/ insecticide	Ag Census; 1978, 1982, 1987, 1992, 1997, 2002, 2007, 2012
**pctc**	% of total acres planted with corn	USDA NASS
**pctsb**	% of total acres planted with soybeans	USDA NASS
**pctw**	% of total acres planted with wheat	USDA NASS
**pctsf**	% of total acres planted with sunflowers	USDA NASS
**pcthay**	% of total acres harvested with hay (incl. alfalfa)	USDA NASS/ includes alfalfa

**Table 2:** Summary statistics of key variables used in the empirical analysis.

Variable	Statistics			
	mean	st dev	Min	Max
**pcthay**	0.151	0.083	0.025	0.490
**pctc**	0.269	0.137	0	0.723
**pctsb**	0.172	0.152	0	0.502
**pctsf**	0.021	0.040	0	0.249
**pctw**	0.150	0.136	0	0.522
**pcti**	0.102	0.077	0	0.449

Note: st dev refers to standard deviation.

The data in Table 3 that show county-level averages for the proportion of acres planted, acres treated with insecticides, and the proportion of counties with aphid infestations for each of the eight Census years, and the parameter estimates from the base and expanded interaction term models are used to estimate the effect of corn and soybean acres as a proportion of total crop acres on the proportion of acres treated with insecticides for 2007 and 2012.

**Table 3:** Eastern SD Census data. Summary statistics for 44 counties—proportion of acres to total cropland: crops, insecticide, and aphids.

Year	Hay	Wheat	PCTI	Corn	Soybeans	Aphid
**1978**	0.191	0.144	0.093	0.242	0.033	0.000
**1982**	0.167	0.165	0.083	0.246	0.067	0.000
**1987**	0.135	0.149	0.081	0.212	0.105	0.000
**1992**	0.139	0.195	0.054	0.266	0.165	0.000
**1997**	0.162	0.162	0.057	0.255	0.228	0.000
**2002**	0.160	0.125	0.075	0.293	0.284	0.450
**2007**	0.122	0.159	0.181	0.347	0.234	1.000
**2012**	0.098	0.066	0.199	0.369	0.301	0.909

Source: Authors’ compilation.

## Results

### Base model estimates

As per the results from the base model, corn, both Census dummy variables, and the aphid interaction term are highly statistically significant and positive (Table 4). The sunflowers coefficient is positive but barely significant (p-value = .1076). A positive coefficient indicates that a unit increase in the fixed effects variable is associated with an increase in the proportion of acres treated with insecticide at the county level. The estimates for soybeans, hay, and wheat are negative and statistically significant. However, the soybean coefficient must be interpreted in conjunction with the soybean*aphid interaction term, which is positive and significant. For 2007, the proportion of soybean acres is 0.234 and the aphid infestation rate is 100% of all counties, so the estimated effect on PCTI is: -0.2104*0.234 + 0.1425*1 = 0.093. In 2012, the proportion of soybeans acres was 0.30, and aphid infestation rate was .909, so the estimated effect on PCTI is -0.2104*0.30 + 0.1425*0.91 = 0.0665 for 2012. The base model empirical estimates of the overall contribution to the extensive use of insecticide is positive for Census years 2007 and 2012. The corn parameter estimate indicates a 1% increase in corn acres planted out of total cropland acres is associated with an estimated 0.184% increase in the share of acres treated with insecticides at the eastern South Dakota county level, *ceteris paribus*.

**Table 4:** Results from base model.

Variable	Estimate	Error	t-value	Pr > |t|
**Intercept**	0.0967	0.012	8.19	<.0001
**CensDum07**	0.0745	0.009	7.75	<.0001
**CensDum12**	0.0779	0.011	6.68	<.0001
**pctc**	0.1836	0.035	5.87	<.0001
**pctsb**	-0.2104	0.020	-10.52	<.0001
**pctsf**	0.1079	0.067	1.62	0.1076
**pcthay**	-0.1700	0.044	-3.81	0.0002
**pctw**	-0.1106	0.022	-4.91	0.0002
pctsb*aphid	0.1425	0.034	4.12	<.0001

### Expanded interaction model estimates

Table 5 reports the results of the expanded interaction term model. The signs and statistical significance of each crop coefficient are consistent with the base model regression, while the coefficients’ magnitudes are slightly different but within approximately one standard error of the previous regression. The one exception is the coefficient for sunflowers, which is now significant at the 5% level. The parameter estimates for the covariate interaction terms indicate that for sunflowers the proportion of acres planted did not have a statistically significant effect on the proportion of crop acres treated with insecticide in 2007 and 2012 relative to previous census years. For hay, the interaction term for 2007 is negative. The wheat interaction term is statistically significant and positive for 2007 but insignificant for 2012. The wheat coefficient is negative but its interaction coefficient for 2007 is positive, indicating that the decline in wheat production in eastern South Dakota observed in 2007 was associated with a higher rate of increase in acres treated with insecticides than in previous Census years.

**Table 5:** Results from the expanded interaction term model.

Variable	Estimate	Error	t-value	Pr > |t|
**Intercept**	0.1115	0.013	8.44	<.0001
**pctc**	0.1501	0.033	4.47	<.0001
**pctsb**	-0.2087	0.020	-10.41	<.0001
**Pctsf**	0.1228	0.059	2.06	0.0408
**Pcthay**	-0.1866	0.049	-3.75	0.0001
**Pctw**	-0.1373	0.019	-7.02	<.0001
pctsb*aphid	0.1405	0.038	3.65	<.0001
pcthay*CensDum07	-0.1845	0.106	-1.82	0.0709
pctsf*CensDum07	-0.0698	0.210	-0.33	0.7401
pctw*CensDum07	0.2540	0.077	3.28	0.0012
pctc *CensDum07	0.1704	0.043	3.88	0.0001
pctc *CensDum12	0.1469	0.044	3.28	0.0012
pcthay*CensDum12	0.0883	0.204	0.43	0.6652
pctsf*CensDum12	0.1084	0.591	0.18	0.8548
pctw*CensDum12	0.1573	0.237	0.66	0.5077

As mentioned earlier, the data in Table 3 and the parameter estimates in Table 5 are used to estimate the extent to which corn acres comprise the proportion of total crop acres treated with insecticides for 2007 and 2012. For corn, the in-sample predicted contribution of corn plantings to acres treated is the sum of the direct and interaction parameter estimates which are multiplied by the associated proportion of acres planted to total crop acres. For 2007, the estimated contribution of county-level corn acres to the predicted average proportion of acres treated with insecticides is equal to: 0.1501*0.347 + 0.1704*0.347 = 11.1%. Similarly, the 2012 estimate is 10.9%.

In 2007, corn accounted for 34.7% of total crop acres in eastern South Dakota. Based on our analysis, corn accounted for an estimated 11.1% of acres treated with insecticide. In the same year, the proportion of total crop acres treated with insecticides was 18.1%, according to the Census of Agriculture. Hence, these results suggest that corn accounted for 61.3% of acres treated with insecticide in 2007. In 2012, the estimate is 54.7%.

## Discussion

While the results reported above are consistent with [2] that used planted acres and included all 66 South Dakota counties, the current study uses total crop acres, includes only South Dakota’s eastern 44 counties, and adds a Census year of observations. Total crop acres represent a broader USDA definition of cropland than planted acres. Thus, it provides a more conservative estimate of the effect of crop selection on the proportion of acres treated with insecticide at the county level. This more conservative approach estimates the PCTI for 2007 at 18.1% of total crop acres for South Dakota counties east of the Missouri River versus the 20% estimate by [2] for all 66 counties in South Dakota. According to the 2012 Census, total cropland increased by 758 thousand acres and the number of acres treated increased by 300 thousand acres in the 44 eastern South Dakota counties, relative to 2008. The county-level average for the proportion of acres treated was 19.9% in 2012. For the 44 eastern South Dakota counties, the 2012 Census statistics indicate that the extensive use of insecticides increased between the two most recent Census reports in the 44 eastern South Dakota counties.

The only base model coefficient contrasting in sign with [2] is the hay parameter estimate, which may be the result of differences in data used between the current study and [2]. For example, the latter study not only included counties east of the Missouri River but western South Dakota counties as well, and the majority of hay production in South Dakota occurs in the 22 counties located west of the Missouri River. Furthermore, wheat—which has a significant presence in the western part of the State—was not included in the previous study. Although the corn parameter estimate in the base model in this study is lower than the previous study [2], our estimate provides additional evidence that the extensive use of insecticides increased as the proportion of corn acreage increased over the period of analysis. Conversely, the extensive insecticide usage increased as the proportion of hay and wheat acres decreased.

Given that *total crop acres* for a given county generally exceed *acres planted*, the proportion of any given crop reported in Table 3 will be lower relative to using planted acres to estimate crop proportion. However, the earlier study reported an in-sample prediction of corn’s contribution to total acres treated of 63% based on the county average of 28.5% of planted acres for all 66 South Dakota counties [2]. This predicted value is two percentage points higher than the current estimate of 61.3% for the 44 eastern South Dakota counties.

While the literature finds that the adoption of Bt crop varieties reduced the usage of insecticides in terms of pounds per acre, the Census data indicate that the extensive usage of insecticide increased between 1996 and 2012. It should be noted that the Census data do not include neonicotinoid seed treatments associated with GM seed varieties when reporting data on acres treated with insecticide. In light of the confines of the Census data and because an estimated one-third of all soybean acres and 79% of all corn acres planted in 2011 were treated with neonicotinoids in the U.S. [9], our results provide a very conservative estimate of the extensive insecticide use in South Dakota. Our results show that the coefficients of the Census dummy variables for 2007 and 2012 were statistically significant and positive and that the estimated increases in total acres treated in 2007 and 2012 were 7.4% and 7.8% higher, respectively, than for the previous Census years included this study. These results reflect the noticeable increase in acres treated for the two most recent Census years, as shown in Fig 2.

Regardless of the estimation differentials between the current and the previous studies, the empirical findings confirm a large increase in the extensive use of insecticides, coinciding with the large increase in corn and soybean acres during the two most recent Census years relative to previous Census years. The increase in the share of acres treated also coincided with an increase in GM crop adoption rates in South Dakota, from 43% in 2002, to 59% in 2007, and to 71% in 2012. Between 2007 and 2012, the county-based average corn acreage increased from 34.7% to 36.9% of total cropland acres, so an estimated 20.5% and 26.2% of corn acres were planted with Bt corn, respectively, in these two years. This implies that non-Bt crop acres in 2007 and 2012 made up of 14.2% and 10.7% of total crop acres planted, respectively. The estimated proportions of acres treated at the county level in 2007 and 2012 planted with corn were 11.1% and 10.9%, respectively. These two sets of estimates suggest that in 2007, 78% (10.9/11.1) and 102% (10.9/10.7) of non Bt corn acres were treated with insecticide in 2007 and 2012, respectively. This suggests some prophylactic spraying of insecticide on Bt corn acres may have occurred in the 44 eastern South Dakota counties.

The diminished complexity of crop rotations practices in the Midwest in general and South Dakota in particular has increased the reliance on mono-cropping. This shift in crop production practices was facilitated by the rapid adoption of GM technology. Unintended consequences of this shift in crop production practices were an increase in use of herbicides and a reduction in insecticide usage in terms of average pounds per acre applied. While the intensive use of insecticides (measured in terms of average pounds per acre applied) has declined [6–8], the extensive use of insecticides (measured by the proportion of crop acres treated) has increased [3, 9] since the introduction of the GM technology in the 1990s.

## Conclusions

The data presented suggest that South Dakota experienced an extensification of insecticide use in the 2007 and 2012 Census reporting years relative to previous Census years. Also, the results empirically link the increase in acres treated with insecticides at the county level in eastern South Dakota to the extensification of insecticide use for specific crops.

USDA data on acres treated with insecticides suggest that other Midwestern states experienced similar increases in recent years. Empirical evidence further indicates that growth in corn acres planted increases the proportion of acres treated with insecticides in South Dakota. Also, as GM crop adoption rose in South Dakota, so did the proportion of acres treated, indicating that producers may have resorted to prophylactic spraying to protect against non-targeted insect pests. While broad-spectrum products may protect against a variety of potential threats, they can also pose potential environmental harm to beneficial species. Furthermore, producers may rely on insecticides to control pests more heavily now than under traditional crop rotations, thus increasing the number of acres treated. An increase in the extensification of insecticide use may have negative implications on the overall-ecosystem health in the long-term, an aspect often overlooked by producers, agribusiness industry, and policy makers.

## Supporting information

S1 Table Variance components statistics and LML model fit statistics for base model (Eq 6). (DOCX)

S2 Table Variance components statistics and LML model fit statistics for interaction model (Eq 7). (DOCX)

S1 File SAS code. (DOCX)
