# Supplementary material for: Extensive usage of insecticide and changing crop rotation patterns: A South Dakota case study
Source: PLoS One. 2018 Nov 29;13(11):e0208222. doi: 10.1371/journal.pone.0208222 (PMC6264870; doi:10.1371/journal.pone.0208222)
Supplement: S1 Table — (DOCX) [file pone.0208222.s001.docx]

**S1 Table. Variance Components Statistics and LML Model Fit Statistics for Base Model (Eq. 6)**

| Covariance Parameter | Covariance Parameter Estimate  & Z statistic |
| --- | --- |
| Intercept | 0.0005: Z=3.06 |
| CensDum07 | 0.0009: Z=1.82 |
| CensuDum12 | 0.0020: Z=2.93 |
| Aphid | 0.0017: Z=3.03 |
| AR(1) | 0.1457: Z=1.60 |
| Residual | 0.0007: Z=8.78 |
| LML Fit Statistics |  |
| -2 Log Likelihood | -1293.1 |
| AIC | -1281.1 |
| BIC | -1270.4 |
| Likelihood Ratio Test: Unrestricted Model (Mixed Effects Versus Restricted Model - Fixed Effects Only) | Likelihood Ratio Test Statistic = 99.0  Pr > ChiSq < .01 with DF = 4. |
