# Supplementary material for: Extensive usage of insecticide and changing crop rotation patterns: A South Dakota case study
Source: PLoS One. 2018 Nov 29;13(11):e0208222. doi: 10.1371/journal.pone.0208222 (PMC6264870; doi:10.1371/journal.pone.0208222)
Supplement: S2 Table — (DOCX) [file pone.0208222.s002.docx]

**S2 Table. Variance Components Statistics and LML Model Fit statistics for Interaction Model (Eq. 7).**

|  | |
| --- | --- |
| Covariance Parameter | Covariance Parameter Estimate  & Z statistic |
| Intercept | 0.0005: Z=3.01 |
| CensDum07 | 0.0011: Z=1.99 |
| CensDum12 | 0.0023: Z=2.87 |
| Aphid | 0.0015: Z=2.86 |
| AR(1) | 0.1582: Z=1.69 |
| Residual | 0.0007: Z=8.65 |
| LML Fit Statistics |  |
| -2 Log Likelihood | -1287.1 |
| AIC | -1275.1 |
| BIC | -1264.4 |
| Likelihood Ratio Test: Unrestricted Model (Mixed Effects Versus Restricted Model - Fixed Effects Only) | Likelihood Ratio Test Statistic=100.3  Pr> ChiSq <.01 with DF=4. |
