# Supplementary material for: Extensive usage of insecticide and changing crop rotation patterns: A South Dakota case study
Source: PLoS One. 2018 Nov 29;13(11):e0208222. doi: 10.1371/journal.pone.0208222 (PMC6264870; doi:10.1371/journal.pone.0208222)
Supplement: S1 File — (DOCX) [file pone.0208222.s003.docx]

**S1 File. SAS Code.**

***NOTE: The Data set imported below is Eastriver2017. This is the data provided at the Harvard Dataverse site: Fausti, Scott, 2018, "Extensive usage of insecticide and changing crop rotation patterns: A South Dakota case study", <https://doi.org/10.7910/DVN/MAY4YN>, Harvard Dataverse, V2.22.

***NOTE: If there are any questions on the code please contact Scott Fausti at [Sfausti@csumb.edu](mailto:Sfausti@csumb.edu) ****;

title 'PLOS paper (Extensive usage of insecticide and changing crop rotation patterns: A South Dakota case study): SD Insecticide Program 2017: has been updated';

***the data is Census Insecticide data.EastRiver2015**

This data set had two west river counties in it and

it left our Brule county in east river. The problem has been corrected. ** ;

**proc** **means** data=Eastriver2017;

**run**;

**data** a;

set Eastriver2017;

************ yearly acres treated ****data multiplied by 8 because all years used but yearly dummies eliminate non year referecne data*

This was done to generate correct mean values PCTI for each Census year for discussion and Table 7.***;

q78INAC=yr78d*pcti***8**;

q82INAC=yr82d*pcti***8**;

q87INAC=yr87d*pcti***8**;

q92INAC=yr92d*pcti***8**;

q97INAC=yr97d*pcti***8**;

q02INAC=yr02d*pcti***8**;

q07INAC=yr07d*pcti***8**;

q12INAC=yr12d*pcti***8**;

***lagTAC=lag(totcropacr)

Need to do this manually because of the cross section nature of the data

difTotAC= totcropacr-lagtac***;

***proc print data=a,

var county countycode,

run, if or 15 or 31 or 33 or 41 or 47 or 53 or 55 or 63 or 71 or 75 or 81 or 85 or 93 or 95 or 103 or 105 or 113 or 117 or 121 or 123 or 137 then delete*

yr82d yr87d yr92d yr97d yr02d yr07d yr12d**;

**run**;

**data** b;

set a ;

if yrcode>**6** then poldum=**1**;

else poldum=**0**;

if yrcode>**7** then poldum12=**1**;

else poldum12=**0**;

if **6**<yrcode<**8** then poldum07=**1**;

else poldum07=**0**;

corndum=pctc*poldum;

if yrcode="." then delete;

if countycode=**19** then delete;**west river counties**;

if countycode=**55** then delete;

if poldum12=**1** or poldum07=**1** then dumpol=**1**;

else dumpol=**0**;

fred=pctc*pctsb*poldum07;

fred2=pctc*pctsb*poldum12;

**proc** **means** data = b;

**run**;

***Random intercept model not as robust with or without noint***;

************cut off***;

title '2007 insecticide rates';

**data** c;

set b ;

if poldum07=**0** then delete;

**proc** **means** data=c;

**run**;

title '2012 insecticide rates';

**data** d;

set b ;

if poldum12=**0** then delete;

**proc** **means** data=d;

**run**;

title '1978 insecticide rates';

**data** e;

set b ;

if yr78d=**0** then delete;

**proc** **means** data=e;

**run**;

title '1982 insecticide rates';

**data** f;

set b ;

if yr82d=**0** then delete;

**proc** **means** data=f;

**run**;

title '1987 insecticide rates';

**data** g;

set b ;

if yr87d=**0** then delete;

**proc** **means** data=g;

**run**;

title '1992 insecticide rates';

**data** h;

set b ;

if yr92d=**0** then delete;

**proc** **means** data=h;

**run**;

title '1997 insecticide rates';

**data** i;

set b ;

if yr97d=**0** then delete;

**proc** **means** data=i;

**run**;

title '2002 insecticide rates';

**data** j;

set b ;

if yr02d=**0** then delete;

**proc** **means** data=j;

**run**;

title 'P model with random policy var and random INT test model Tia data';

**proc** **mixed** data=b covtest empirical; *******ML was used so we could do model chi sq selection test******;

class countycode yrcode;

model pcti= poldum07 poldum12 pctc pctsb pctsf pcthay pctw pctsb*aphid / s chisq outpred=P;

random int poldum07 poldum12 aphid/ subject=countycode s;

repeated yrcode / type=AR(**1**) subject=countycode ;

**run**;

**proc** **corr** data=P;

var poldum pctc pctsb pctsf pcthay pctw aphid resid pcti ;

**run**;

**proc** **means** data=b;

**run**;

title 'Corn Model New: 2012 and 2007 interaction terms term in G model with no policy dummies';

**proc** **mixed** data=b covtest empirical; *******ML was used so we could do model chi sq selection test******;

class countycode yrcode;

model pcti= pctc pctsb pctsf pcthay pctw pcthay*poldum07

pctc*poldum07 pctsf*poldum07 pctw*poldum07 pcthay*poldum12

pctc*poldum12 pctsf*poldum12 pctw*poldum12 pctsb*aphid/ s cl chisq outpred=tt;

random int poldum07 poldum12 aphid/ type=vc subject=countycode ;

repeated yrcode/ type=ar(**1**) subject=countycode ; ;

**run**;
